# Editorial: Gut and circulating microbiota in the pathophysiology and clinical complications of diabetes

**DOI:** 10.3389/fcdhc.2025.1717638

**Published:** 2025-10-10

**Authors:** Dinakaran Vasudevan, Velmurugan Ganesan, Chaitanya Dende, Chethan Sampath

**Affiliations:** ^1^ Gut Microbiome Division, Scientific Knowledge on Aging and Neurological Ailments (SKAN) Research Trust, Bengaluru, India; ^2^ Chemomicrobiomics Laboratory, Department of Biochemistry and Microbiology, KMCH Research Foundation, Coimbatore, India; ^3^ Central Research Laboratory, KMCH Institute of Health Science & Research, Coimbatore, India; ^4^ Department of Immunology, University of Texas Southwestern Medical Center, Dallas, TX, United States; ^5^ Department of Oral Diagnostic Sciences & Research, School of Dentistry, Meharry Medical College, Nashville, TN, United States

**Keywords:** gut microbiota, circulating microbiota, diabetes mellitus, short-chain fatty acids, microbiome diversity, oral virome, diabetic kidney disease, enteric nervous system

## Introduction

Diabetes mellitus is a multifactorial metabolic disorder characterized by chronic hyperglycemia and progressive multisystem complications. Growing evidences positions the microbiome (including intestinal microbial communities comprising of bacteria, viruses, fungi and protozoans and their metabolites) as one of the major axes of disease pathogenesis alongside host genetics and life style factors. Beyond change in gut microbial diversity, dysbiosis leads to disturbance of gut barrier integrity, trigger immune activation, and alter metabolic signaling through short-chain fatty acids (SCFAs), bile acids, and tryptophan metabolites, as well as epigenetic regulation (1, 2). Circulating microbial DNA and metabolites are increasingly investigated as potential biomarkers for disease progression and complications (3).

This Research Topic, Gut and Circulating Microbiota in the Pathophysiology and Clinical Complications of Diabetes, gathers seven contributions that explore how gut and circulating microbiota shape diabetes pathophysiology and clinical complications, from microbial metabolites and viral elements to organ specific outcomes.

## Pathophysiology and clinical complications of diabetes

Diabetes arises from impaired insulin secretion, insulin resistance, or both, with contributions from genetic predisposition, environmental triggers, and systemic inflammation. These drive β-cell dysfunction, oxidative stress, and dysregulated glucose and lipid metabolism (4). Chronic hyperglycemia promotes formation of advanced glycation end-products (AGEs), causing vascular damage and endothelial dysfunction (5), which in turn underlie microvascular complications (retinopathy, nephropathy, neuropathy) and macrovascular complications (atherosclerosis, coronary artery disease, stroke) (6). Low-grade inflammation and altered gut and circulating microbiota amplify metabolic dysregulation, further increasing risks of cardiovascular and hepatic disease (2). Together, these mechanisms explain the broad clinical spectrum that makes diabetes a global health burden.

## Gut and circulating microbial components

The gut microbiota exerts both local and systemic effects through the release of microbial products into circulation. These include cell-free microbial DNA (cf-mDNA), lipopolysaccharide (LPS), peptidoglycans, extracellular vesicles, and metabolites such as SCFAs, bile acids, and trimethylamine N-oxide (TMAO) (7). Gut barrier dysfunction facilitates the translocation of these components, which interact with host receptors such as Toll-like receptors (TLRs) and NOD-like receptors to modulate immune and metabolic pathways (1, 2). Elevated circulating microbial DNA and endotoxin levels have been associated with metabolic disorders, cardiovascular disease, and sepsis, suggesting potential as biomarkers (3). SCFAs improve glucose homeostasis and inflammation, whereas TMAO and secondary bile acids promote cardiometabolic complications (8). Thus, circulating microbial components function as both mediators of host–microbe interactions and candidate biomarkers for precision medicine.

## Microbial components in diabetes

Microbial products, including cf-mDNA, LPS, peptidoglycans, and metabolites, are implicated in diabetes pathogenesis. Obesity-associated gut barrier dysfunction enables their translocation, driving “metabolic endotoxemia” (9). Circulating LPS engages TLR4 signaling, inducing chronic inflammation and insulin resistance (10). Similarly, altered microbial DNA signatures have been reported in diabetic patients, pointing to biomarker potential (3).

Microbial metabolites also play dual roles: SCFAs confer metabolic and anti-inflammatory benefits, whereas TMAO and certain bile acids exacerbate cardiovascular risk (8). These findings support the view that microbial components are active drivers of immune–metabolic crosstalk in diabetes.

## Highlights of the Research Topic

### Gut-associated metabolites and diabetes pathology

Gough et al. systematically reviewed 34 studies across type 1 diabetes (T1DM), gestational diabetes (GDM), prediabetes, and type 2 diabetes (T2DM), identifying 272 metabolites from 38 classes associated with diabetes. SCFAs and bile acids emerged repeatedly, but considerable methodological heterogeneity (sample type, platforms) limited comparability.

### Duodenal mucosal resurfacing with GLP-1RA in T2DM

Meiring et al. studied 16 insulin-treated T2DM patients undergoing duodenal mucosal resurfacing (DMR) combined with GLP-1 receptor agonists (GLP-1RA). Clinical improvements included insulin withdrawal in many patients. Importantly, increases in α-diversity correlated with HbA1c reduction, and β-diversity changes correlated with liver fat fraction improvements.

### Gut microbiome in obese Mongolians with and without T2DM

Shinoda et al. explored the “Mongolian paradox,” comparing obese individuals with and without T2DM. Nondiabetic obese participants had higher abundances of SCFA producers (*Anaerostipes hadrus*) and faecal acetate, whereas diabetic obese individuals had pro-inflammatory taxa (*Methanobrevibacter*, *Desulfovibrio*) and reduced *Faecalibacterium*. SCFA levels were among the strongest negative associations with diabetes.

### Maternal Western diet, microbiome, and fetal programming

Sugino et al. demonstrated in a baboon model that maternal Western-style diet altered the maternal gut microbiome and induced changes in placental and fetal hepatic miRNA/gene expression. This work highlights maternal diet–microbiome interactions as drivers of developmental programming of metabolic disease risk.

### Oral virome in diabetes

Zhang et al. expanded the microbiome perspective by characterizing the oral virome in patients with diabetes. Distinct viral community structures, altered virus–bacteria interaction networks, and functional pathway shifts were observed, underscoring the role of viral components in diabetic dysbiosis.

### Microbiota and enteric nervous system crosstalk in diabetic gastroenteropathy

Tao et al. reviewed evidence linking gut dysbiosis to enteric nervous system (ENS) dysfunction in diabetic gastroenteropathy. They outlined how microbial metabolites, hormones, and neurotransmitters interact with the ENS, framing the microbiota–ENS axis as a therapeutic target for gastrointestinal complications.

### Gut microbiota in diabetic kidney disease

Wu et al. compared gut microbiota across DKD, long-term diabetes without nephropathy, and DKD with non-diabetic renal disease. While overall diversity was similar, taxa such as *Olsenella* were enriched and *Faecalibacterium prausnitzii* depleted in DKD. Distinct functional predictions (e.g., pyruvate metabolism) may help explain renal involvement and serve as potential biomarkers.

## Shared insights and implications

Across these studies, several unifying themes emerge:

SCFA producers confer protective effects (Gough et al.; Shinoda et al.; Wu et al.).

Microbial diversity correlates with metabolic improvement and complication profiles (Meiring et al.; Shinoda et al.).

Expansion beyond bacteria to viruses (Zhang et al.), ENS interactions (Tao et al.), and epigenetics (Sugino et al.) enriches mechanistic understanding.

Organ-specific microbiota signatures (Wu et al.; Tao et al.) link dysbiosis to complications such as DKD and gastroenteropathy.

## Conclusion

Together, these contributions underscore the central role of gut and circulating microbiota in diabetes. They highlight the protective role of SCFA-producing taxa, the impact of diet and interventions on microbial diversity, and the expanding importance of non-bacterial elements and host–microbiota crosstalk. Future progress will require longitudinal and interventional studies, methodological standardization, and integration of multi-omics approaches. Translating these findings into diagnostics and targeted therapies promises to advance precision medicine for diabetes and its complications.
